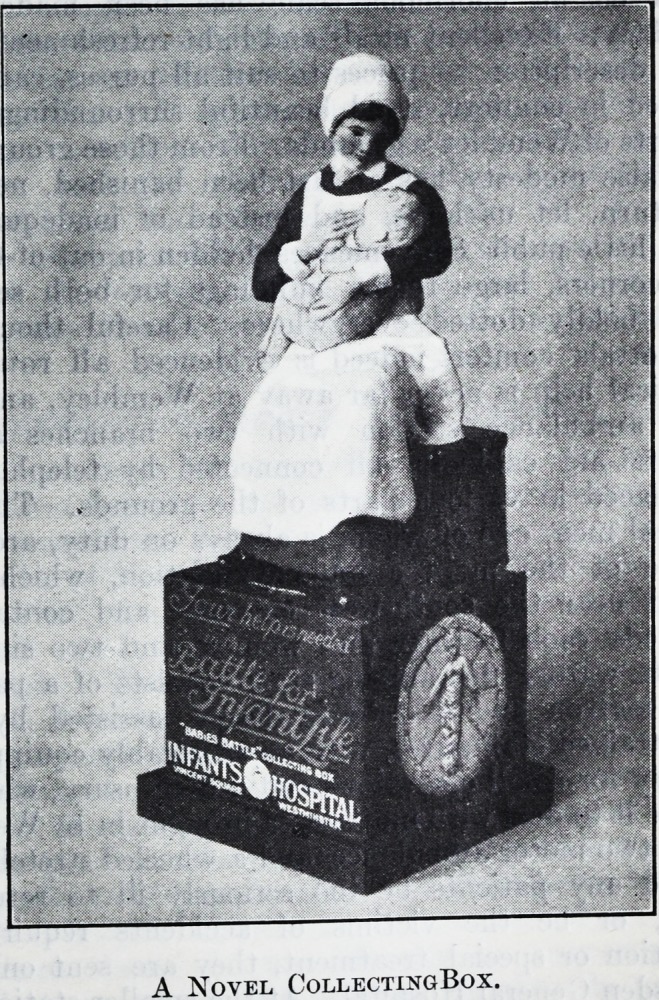# Please!

**Published:** 1924-06

**Authors:** 


### " PLEASE ! "

This charming collecting box has been designed and made for the Infants' Hospital at Vincent Square in the hope that those who see it on shop counters will be unable to resist dropping a coin into it to

A Novel CollectingBox.

enable the little blue-clad nurse to help her chubby charge to retain his chubbiness. They will do so with the knowledge that they are helping a staff of nurses to save from death or invalidism hundreds of suffering children.

## Figures and Tables

**Figure f1:**